# *Cacna1c* Hemizygosity Results in Aberrant Fear Conditioning to Neutral Stimuli

**DOI:** 10.1093/schbul/sbz127

**Published:** 2020-01-07

**Authors:** Anna L Moon, Nichola M Brydges, Lawrence S Wilkinson, Jeremy Hall, Kerrie L Thomas

**Affiliations:** 1 Neuroscience and Mental Health Research Institute, Cardiff University, Cardiff, UK; 2 MRC Centre for Neuropsychiatric Genetics and Genomics, Cardiff University, Cardiff, UK; 3 School of Psychology, Cardiff University, Cardiff, UK; 4 School of Biosciences, Cardiff University, Cardiff, UK

**Keywords:** calcium, learning, fear, salience, animal models, psychosis

## Abstract

*CACNA1C,* a gene that encodes an alpha-1 subunit of L-type voltage-gated calcium channels, has been strongly associated with psychiatric disorders including schizophrenia and bipolar disorder. An important objective is to understand how variation in this gene can lead to an increased risk of psychopathology. Altered associative learning has also been implicated in the pathology of psychiatric disorders, particularly in the manifestation of psychotic symptoms. In this study, we utilize auditory-cued fear memory paradigms in order to investigate whether associative learning is altered in rats hemizygous for the *Cacna1c* gene. *Cacna1c* hemizygous (*Cacna1c*^*+/−*^) rats and their wild-type littermates were exposed to either delay, trace, or unpaired auditory fear conditioning. All rats received a Context Recall (24 h post-conditioning) and a Cue Recall (48 h post-conditioning) to test their fear responses. In the delay condition, which results in strong conditioning to the cue in wild-type animals, *Cacna1c*^*+/−*^ rats showed increased fear responses to the context. In the trace condition, which results in strong conditioning to the context in wild-type animals, *Cacna1c*^*+/−*^ rats showed increased fear responses to the cue. Finally, in the unpaired condition, *Cacna1c*^*+/−*^ rats showed increased fear responses to both context and cue. These results indicate that *Cacna1c* heterozygous rats show aberrantly enhanced fear responses to inappropriate cues, consistent with key models of psychosis.

## Introduction

Genetic variation in *CACNA1C*, a gene that encodes the pore-forming alpha-1 subunit of Ca_V_1.2 L-type voltage-gated calcium channels (LTCCs), has been strongly and consistently linked to both schizophrenia and bipolar disorder, among other psychiatric disorders.^1–3^ While schizophrenia and bipolar disorder can present very differently in the clinic, both are associated with a psychosis phenotype,^4^ and several studies have indicated that there is a shared genetic architecture between the two disorders,^5,6^ including in *CACNA1C.*^7^

Studies have shown the altered expression of *CACNA1C* in individuals with *CACNA1C* common risk variants, mostly decreased expression,^8–10^ although some studies have indicated an increased expression in certain brain regions.^11^ Although most studies have concentrated on common variations, rare variants in *CACNA1C* have also been implicated in psychiatric disorders.^12,13^

Calcium signaling in neurons is responsible for regulating neuronal excitability, synaptic plasticity, cognition and information processing, and features considered to be impaired in psychiatric disorders.^14^ Therefore, there has been substantial interest in the role of Ca_v_1.2 on endophenotypes associated with these disorders. Altered associative learning has been implicated in the pathology of various psychiatric disorders,^15–20^ particularly in the manifestation of positive (psychotic) symptoms.^21^ There is also evidence at a genomic level that these learning processes are implicated in risk for schizophrenia.^13,22,23^

One powerful method of studying associative learning is the use of tightly controlled Pavlovian fear conditioning. Indeed, associative learning in the context of Pavlovian fear has been shown to be altered in patients with schizophrenia.^24^ Aversive conditioning in schizophrenic populations has been shown to result in abnormal retrieval of safety cues and reduced extinction, culminating in increased learnt fear responses in these patients.^25,26^ Pavlovian fear conditioning involves the pairing of a neutral conditioned stimulus (CS) with an unconditioned stimulus (US). The CS–US associations formed evoke a conditioned response upon subsequent presentations of the CS alone.^27^ The exact relationship between the presentation of CS and US determines what associations are formed. Subtle manipulations of the contingencies between the CS and US can lead to significant differences in the associations formed, as exemplified by trace and delay conditioning. During delay conditioning, CS and US are presented together and a strong association between the two is formed.^28^ In trace conditioning, CS and US are separated by a temporal gap, the trace interval, between CS cessation and US onset. This results in weaker CS–US associations and increased conditioning to contextual features.^29^ As the trace interval increases, conditioning to the CS decreases,^27^ and there is increased conditioning to the contextual stimuli.^30^ While both delay and trace fear conditioning depend on activity in the amygdala, trace conditioning relies on additional circuity,^31^ including the hippocampus and prefrontal cortex, reflecting their roles in encoding the multimodal features of an environment^32^ and temporal information.^33^

Dysfunction in the encoding of associations by brain structures such as the hippocampus and prefrontal cortex has been hypothesized to contribute to the development of psychosis.^34^ One prevailing theory holds that psychosis derives from an increased response to irrelevant stimuli, also known as aberrant salience.^35–38^ Aberrant salience represents the inclination to inappropriately “tag” neutral or irrelevant cues with importance,^35^ leading to inappropriate associations being formed.^39^ It has been reported that patients with schizophrenia present with an inability to ignore irrelevant stimuli.^38,40,41^ It is argued that the assignment of importance to inappropriate cues can, over time, contribute to the formation and maintenance of psychotic symptoms.^21,36,38^ Therefore, how associations are created and maintained during learning is important when considering the development of psychopathology.

Previous studies have suggested that the total loss of forebrain *Cacna1c* impacts on associative learning processes,^42–44^ although this is not consistent across studies.^45,46^ However, the genetic variants in *Cacna1c* associated with schizophrenia and related disorders do not produce a total loss of the gene, but instead likely influence the expression.^9–11^ In this study, we investigate the responses of *Cacna1c* hemizygous rats after delay, trace, and unpaired contextual fear conditioning in order to determine the role of dosage of Ca_v_1.2 on associative fear memory. We specifically sought to determine whether altered dosage of *Cacna1c* impacts on conditioning to less-salient cues in associative fear learning paradigms.

## Methods

### Animals

Adult male *Cacna1c* hemizygous (*Cacna1c*^*+/−*^) rats on a Sprague Dawley background (TGR16930, Horizon, Sage Research Labs, USA) and wild-type littermates were obtained and housed in mixed-genotype groups of 2–4 in standard cages (38cm × 56cm × 22cm) with *ad libitum* access to food and water. This model is a constitutive zinc finger nuclease knockout, resulting in an approximately 50% and 40% decrease in hippocampal mRNA and protein levels, respectively, with diseases in other brain regions such as the prefrontal cortex.^47^ Therefore, this model accords with reduced brain expression in *CACNA1C* in patient cohorts.^9,10^ All animals were housed on 12:12h light–dark cycles. Experiments were conducted in accordance with local ethics guidelines, the UK Animals (Scientific Procedures) Act 1986 and the European Communities Council Directive (1986/609/EEC).

### Auditory Fear Conditioning

**Fig. 1.**
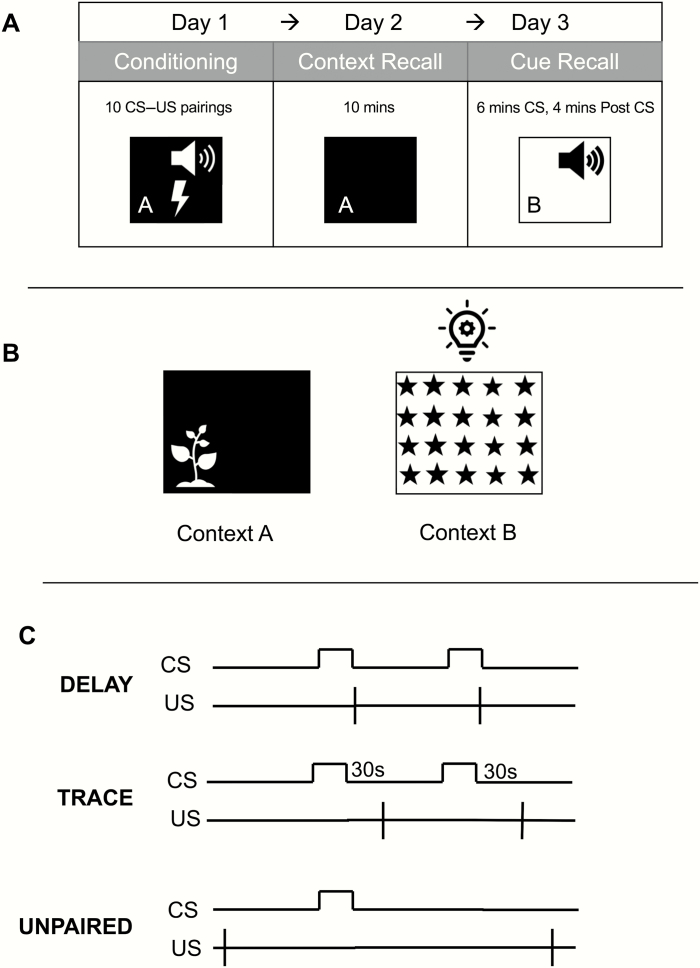
Experimental overview for fear conditioning and test sessions. A = Rats were conditioned on Day 1 in Context A or B in either a delay, trace, or unpaired paradigm. They received a 10-min Context Recall on Day 2 where they were returned to their Day 1 context for 10 min. On Day 3, rats were placed into a novel context and presented with the auditory cue during a Cue Recall session. B = Two contexts were used to distinguish the behavioral reactions to the context and auditory cue. Context A was a darkened conditioning chamber (houselight off) with a lavender scent, whereas Context B was a lit chamber (houselight on) with a starred background wallpaper and sawdust. C = Delay, trace, and unpaired conditioning schematic. In delay conditioning, the auditory CS co-terminates with the US. In trace conditioning however, the US is presented 30 s after the termination of the CS. In unpaired conditioning, the CS and US were explicitly unpaired.

Animals were trained in either delay, trace, or unpaired fear conditioning at PND 60–70 within the light cycle period. In total, 44 animals were used (22 wild-types, 22 *Cacna1c*^*+/−*^, weight 350–500 g), with 7–8 per genotype used per protocol. Animals were placed into one of two standard rat conditioning chambers (Supplementary S2). Two contexts were used for the analysis (Supplementary S2, figure 1); 15 s of 75-6db white noise formed the CS and the US was 0.5 s, and 0.5 mA scrambled footshock in all protocols. The use of the two contexts was counterbalanced across genotype. Behavior was recorded by a digital video camera. Rats were habituated for three days prior to the conditioning session by being transported to the testing room once a day, briefly handled and returned to their home cages.

#### Conditioning and Recall.

Rats were conditioned using a protocol previously established by our group.^[^48^]^ Rats were individually placed in either context A or B in a counterbalanced manner and given a 120-s baseline habituation period (Baseline). For delay conditioning, animals were presented with 10 CS–US pairings separated by an intertrial interval (ITI) of 312+/−62s, where the CS co-terminated with the US. For trace conditioning, rats also received 10 CS–US pairings separated by an ITI of 312+/−62s; however, the CS and US were separated by a 30-s trace period between the offset of the CS and onset of the US. This trace period has previously been shown to be an optimal length for trace conditioning.^[^49,50^]^ For unpaired conditioning, rats received 10 presentations of the CS and 10 presentations of the US in an explicitly unpaired manner (CS–US interval = 156+/−31s), separated by an ITI of 312+/−62s. Twenty-four hours post-conditioning, rats were returned to the chamber they were conditioned in for 10 min in the absence of both CS and US to assess contextual fear responses (Context Recall). Two days post-conditioning, rats were placed in the nonconditioned chamber. The nonconditioned context was further altered by offsetting the time of day tested by 3 h and by placing a plastic insert over the shock bars. A 120-s baseline period occurred (Novel Baseline) before a 6-min CS presentation to examine CS-evoked fear responses (CS). Following CS cessation, animals were monitored for a further 4 min (Post-CS).

#### Analysis.

Fear responses were measured by freezing behavior. Freezing behavior was defined as complete immobility for 1 s except for movement required for respiration. Animals were scored every 10 s, with the experimenter blind to conditions. The percentage (%) of time freezing was calculated for each animal for each period during each training (Pre-US and Post-US) and test session (Context Recall, and Novel Baseline, CS, and Post-CS during Cue Recall). A model was set up for each condition (Delay, Trace, and Unpaired) separately as they were performed on different sets of animals. Two-way repeated measures ANOVAs were set up to analyze acquisition on conditioning day (Baseline vs Post-US). For Context Recall, a one-way ANOVA was used to compare responses between genotypes. Finally, for Cue Recall, a two-way repeated measures ANOVA was performed to analyze responses to each part of the recall test (Novel Baseline vs CS vs Post-CS). Tukey–Kramer honestly significant difference (Tukey–Kramer HSD) *post hoc* tests were performed where appropriate. All data were checked for homogeneity of variance (Levene’s Test) and normality of distribution and transformed by square-root transformations if necessary. Data were analyzed in JMP statistical software (SAS Institute, USA). Results were assumed to be significant if *P* < .05. Graphs were made in GraphPad Prism (Version 7 for Windows, GraphPad Software, La Jolla California USA).

## Results

### Increased Contextual Fear Memory Following Delay Auditory Conditioning was Observed with Cacna1c Hemizygosity

**Fig. 2.**
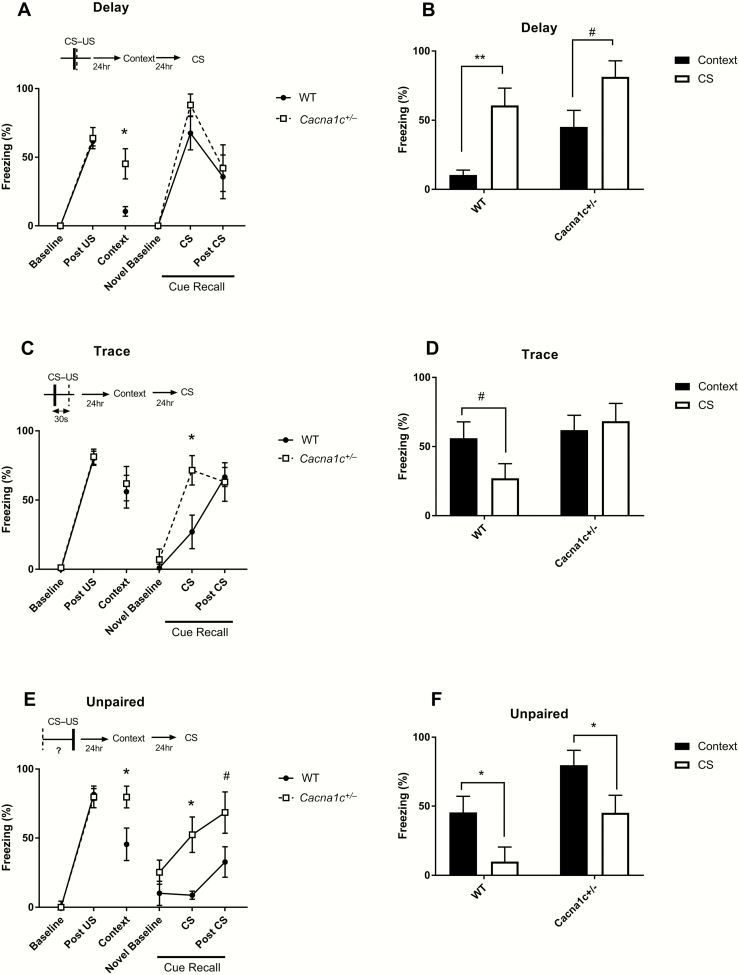
Delay, trace, and unpaired fear conditioning in wild-type Cacna1c^*+/−*^ rats. A = Following delay conditioning, Cacna1c^*+/−*^ rats display increased freezing responses to conditioned context (Context) in comparison to wild-types, although wild-types still freeze more than at baseline. Nevertheless, the Cacna1c^*+/−*^ rats show a similar response to wild-types in a novel context (Novel Baseline) and to the auditory CS during Cue Recall. B = Wild-type rats freeze significantly more to the CS during Cue Recall than to the conditioning context in the Context Recall session after delay conditioning but for the heterozygotes, this difference is smaller (*n* = 7/genotype). C = Following trace conditioning, Cacna1c^*+/−*^ rats show comparable freezing behavior to the wild-types during Context recall; however, they display increased freezing during auditory CS presentation during the Cue Recall session. Similar levels of freezing to wild-types were noted during Novel Baseline and Post-CS periods. D = Trace conditioned wild-type rats show a trend to freezing less to the auditory CS during Cue Recall than to the conditioning context, but for Cacna1c^*+/−*^ rats there is no difference (*n* = 8/genotype). E = After unpaired conditioning, Cacna1c^*+/−*^ rats show increased fear responses to the conditioned context and CS. F = Both wild-type and Cacna1c^*+/−*^ rats freeze more to the conditioned context than the CS (following unpaired conditioning) (*n* = 7/genotype). There were no behavioral differences between the Cacna1c^*+/−*^ and wild-type rats during delay, trace, and unpaired training (Baseline and Post-US periods). Data points represent mean percentage freezing per group for each session; error bars are SEM. Cacna1c^*+/−*^ vs wild-type rats; # = *P* < .1, * = *P* < .05, ** = *P* < .01, Tukey Kramer HSD.

The presentation of the US during conditioning elicited a robust freezing response during delay conditioning in both *Cacna1c*^*+/−*^ and wild-types (*F*_(1,12)_ = 217.472, *P* < .001; figure 2A). There was no difference between genotypes (*F*_(1,12)_ = 0.010, *P* = .923), indicating that a reduction in *Cacna1c* dosage did not affect the perception and behavioral responses to the aversive stimuli during delay conditioning.

During the Context Recall test 24 h later, *Cacna1c*^*+/−*^ rats froze significantly more than wild-types overall in the conditioning context (*F*_(1,12)_ = 6.86, *P* = .028). However, there was no effect of genotype during Cue Recall session in the novel context on Day 3 (*F*_(1,12)_ = 1.481, *P* = .247) and no Session*Genotype interaction (F_(1,12)_ = 1.73, *P* = .136), with both groups showing a large conditioned response to the CS presentation. Therefore, wild-type rats freeze more to the CS than to context, whereas in heterozygotes, this difference is reduced (figure 2B). Thus, while the heterozygous *Cacna1c* rats acquired and retrieved cued fear memory similar to wild-type animals in the Delay conditioning procedure, they showed increased contextual fear associations. Nevertheless, the *Cacna1c*^*+/−*^ rats were able to discriminate between the conditioned and nonconditioned context as no freezing was seen in the novel context (Novel Baseline) during Cue Recall (figure 2A).

### Trace Conditioned Cacna1c^*+/−*^ Rats Show Aberrant Cue Responses

During trace conditioning *Cacna1c*^*+/−*^ and wild-types rats showed an increase in freezing following exposure to the US (*F*_(114)_ = 859.3, *P* < .001) (figure 2C), with no effect of genotype (*F*_(1,14)_ = 0.833, *P* = .377).

*Cacna1c* ^*+/−*^ and wild-type animals showed similar levels of conditioned fear during the Context Recall test on Day 2 (*F*_(1,14)_ = 0.075, *P* = .788). However, at Cue Recall on Day 3, there was an effect of session (*F*_(3,13)_ = 27.94, *P* < .001) with a trend for a genotype effect (*F*_(1,14)_ = 3.281, *P* = .092) and a significant Session*Genotype interaction (*F*_(3,13)_ = 3.870, *P* = .048). *Post hoc* tests revealed that *Cacna1c*^*+/−*^ rats froze significantly more than wild-types during the CS presentation (*P* = .003). Thus after trace conditioning, *Cacna1c*^*+/−*^ rats show levels of conditioned contextual freezing similar to wild-types but increased responses to the auditory cue, such that the fear responses to the conditioned context and cue are indistinguishable (figure 2D).

To determine if the perception of fear of the auditory cue between genotypes may be driving this effect, i.e. there is a difference between the *Cacna1c*^*+/−*^ and wild-types in finding the noise more fearful in itself, we analyzed freezing behavior following CS presentation without an associated footshock. No freezing behavior was observed in either *Cacna1c*^*+/−*^ rats or wild-types, suggesting that the CS is not enough to drive a fear response alone (Supplementary S3).

### Unpaired Auditory Fear Conditioning Results in Increased Fear Memory in Cacna1c^*+/−*^ Rats

Both conditioned *Cacna1c*^*+/−*^ and wild-type rats display similar acquisition of fear memory during unpaired training (*F*_(1,12)_ = 209.173, *P* < .001) (figure 2E), with both genotypes freezing similarly to the shock (*F*_(1,12)_ = 1.702, *P* = .217).

*Cacna1c* ^*+/−*^ rats displayed increased contextual fear memory as shown by higher freezing to the conditioned context on Day 2 than wild-types (*F*_(1,12)_ = 5.030, *P* = 0.045). During Cue Recall, *Cacna1c*^*+/−*^ rats also showed increased conditioning to the unpaired cue. Thus, in the Day 3 cue test, there was a significant effect of Session (*F*_(3,11)_ = 6.632, *P* = .013) and Genotype (*F*_(1, =12)_ = 6.90, *P* = .022) on freezing behavior. *Post hoc* analysis revealed that the genotype effect was accounted for by increased freezing responses in the *Cacna1c*^*+/−*^ rats during auditory cue presentation (*P* = .002). Compared to wild-types, there also was a trend to increased conditioned fear responses during the Post-CS period (*P* = .08). Despite enhanced responding to the auditory cue, *Cacna1c*^*+/−*^ rats showed higher responding to the context than the auditory cue similar to the pattern seen in the wild-types (figure 2F). Together, these results show increased contextual and cued fear memory in *Cacna1c*^*+/−*^ rats.

## Discussion

Wild-type rats show the expected conditioned behavioral responses to the discrete CS, whereby robust freezing behavior was seen when the CS co-terminated with the US (Delay) but decreased when an interval was explicitly interposed during training (Trace and Unpaired).^27^ Within the Unpaired group, conditioned responding to the context was higher than to the CS because the context better predicts the US.^30^ However, in the Trace group, while the CS still predicts the US, the 30-s delay makes encoding this association difficult, allowing the context to also gain salience. The genetic knockdown of *Cacna1c* had significant effects on these paradigms. Following delay conditioning, *Cacna1c*^*+/−*^ rats showed increased contextual fear, whereas in trace fear conditioning they showed increased freezing to CS. Unpaired conditioning resulted in increased fear responses in both recalls. These results suggest that *Cacna1c* heterozygosity results in normally less-salient cues gaining aberrant salience during associative learning tasks.

Initial acquisition of fear memory was unaffected in *Cacna1c*^*+/−*^ rats with all rats displaying robust post-US freezing to the same degree as the wild-types. This is in agreement with previous studies of Ca_V_1.2 homozygous deletion in neuron-specific mouse knockout models which show normal acquisition of auditory^44,46^ and contextual^43^ fear conditioning. Also, *Cacna1c*^*+/−*^ rats responded to the CS in a similar way to wild-types, suggesting that the perception of the sensory properties of the CS and US is maintained. It is also important to note that *Cacna1c*^*+/−*^ rats had no greater baseline fear or anxiety levels, as indicated by the lack of freezing response at Baseline and Novel Baseline. This is in line with previous behavioral analysis performed in this model, that showed no differences in anxiety in an open field test and no basal fear differences in startle response between genotypes.^47^

Cued and contextual fear conditioning is intact in the *Cacna1c*^*+/−*^ rats. In delay conditioning, robust conditioned freezing was seen upon the presentation of the CS in a novel context. Likewise, *Cacna1c* hemizygosity does not impair the acquisition and retrieval of contextual fear memory, both Trace and Unpaired groups show robust freezing responses upon exposure to the training context (Context Recall), but not to a novel context (Novel Baseline, Cue Recall). These data suggest that CS–US associative learning is not diminished in the *Cacna1c*^+/−^ rats. Nevertheless, the *Cacna1c*^+/−^ rats show evidence of inappropriate conditioned freezing to cues that were present during fear memory training but do not elicit such a strong response in wild-types. Thus, the mutant animals show abnormally high responding to the conditioned context after delay conditioning and also to the CS in trace and unpaired training. It is unlikely that generalized fear to contexts underlies this observation because the *Cacna1c*^+/−^ rats can discriminate between the context in which conditioning occurred (high levels of freezing on Day 2) and one that did not (very-low level response at Novel Baseline on Day 3).

It has been proposed that during trace or unpaired conditioning, the interval between CS and US results in the CS signaling the absence of the US. Therefore, the animal learns that the US will not occur when the CS is present.^51,52^ As such, through the formation and recall of an inhibitory CS–no US association, the CS may act as a “safety signal.” Due to the design of our experiments in which the CS was presented in a novel context, we do not have direct evidence that the CS acts to reduce conditioned responses produced by the conditioned context in wild-types.^53^ However, in Cue Recall, lower freezing responses were seen in the presence of the CS than in the Post-CS intervals in wild-type rats after trace and unpaired conditioning. This may be evidence of the CS acting as a safety cue. This observation has been also reported by others using similar experimental procedures to ours.^54,55^ It is notable that the *Cacna1c*^*+/−*^ rats did not show a reduction in freezing in the presence of the CS, which suggests that *Cacna1c* hemizygosity may impair inhibitory CS–no US learning or retrieval.

The high levels of freezing seen to the training context by delay conditioned *Cacna1c*^*+/−*^ rats may also indicate an impairment in the encoding or retrieval of context-no US associations. In delay conditioning, the animal learns an association between the CS and US; however, the US also enters associations with the background context.^30^ As the context is always present, it can gain predictive properties including to signal the absence of the US. The higher than expected freezing behavior in the *Cacna1c*^*+/−*^ rats may indicate a weaker context–no US association to influence the behavioral expression of fear elicited by the context–US memory. It is possible that the context–no US association may be stronger with unpaired than trace conditioning, because the ITI intervals are variable, and the context is more ambiguous in predicting the occurrence of the US.^56^ This view would account for the supramaximal responding in unpaired trained *Cacna1c*^*+/−*^ rats with impaired context–no US memory. Together our data suggest that the *Cacna1c*^*+/−*^ rats show a general deficit in stimulus–no outcome associative encoding or retrieval and therefore that Ca_V_1.2 LTCCs are required for inhibitory memory.

The observed impairments in inhibitory learning may be indicative of a form of aberrant salience of fear memory, with the *Cacna1c* heterozygotes attributing fear to all aspects of conditioning. Psychosis has been suggested to be a result of aberrant salience—an increased focus on neutral stimuli that leads to altered learning and perception.^19,36,38,57^ It has been suggested that positive symptoms in schizophrenia are a result of this fundamental abnormality in learning that leads to neutral or “safe” stimuli being misinterpreted as relevant or important.^38,41^ Therefore, within this context, the results presented in this study may be explained as *Cacna1c* heterozygote rats experiencing aberrant salience; assigning importance to less relevant stimuli within the task leading to abnormal associations being formed. This can be seen in delay and unpaired conditioning as an inappropriate formation of a context–US association, and inappropriate CS–US association in trace and unpaired. As demonstrated by their increased fear responses, these formations can have a large impact on learnt behaviors and responses to stimuli.

Although the biology behind aberrant salience is not fully understood, Kapur proposed that, in psychosis, a patient experiences an increased, and out of synchrony, release of dopamine that leads to a hyperdopaminergic state.^36^ Whilst this study did not investigate dopamine release during conditioning, LTCCs have been shown to be required for normal dopaminergic transmission between the nucleus accumbens and ventral tegmental area,^58–60^ and *Cacna1c* hemizygosity in mice has been shown to result in an attenuated response to a dopamine transporter blocker and a diminished locomotor response to dopamine elevating psychostimulants.^58^ An investigation into the dopamine system in *Cacna1c*^*+/−*^ rats may be beneficial for further interpretation of the current results. Furthermore, there is a potential that the abnormal context-specific effects seen in the *Cacna1c*^*+/−*^ rats may arise from aberrant salience as a consequence of a habituation deficit, where the context does not become familiar and thus attention to it is maintained.^39^ This habituation impairment may generate sensitiation to the stimulus, resulting in the enhanced freezing observed.^39^ This would require further investigation into how these mechanisms interact.

Finally, it should be noted that these experiments were only performed in males. Most preclinical studies in neuroscience use males,^61^ yet females show a higher prevalence of fear and anxiety disorders,^62^ and differ in the development and expression of schizophrenia.^63^ Given that there is now a growing understanding of sex differences in learning and memory, it is imperative to understand the biology in both sexes under these paradigms.^64^

To summarize, *Cacna1c* heterozygote rats consistently demonstrate altered fear responses due to impaired inhibitory learning or retrieval, in the absence of a more general increase in anxiety. This suggests that mechanisms for regulating cue-specific learning are impaired when *Cacna1c* dosage is altered, leading to aberrant attribution of salience to inappropriate cues. Such altered associative learning may contribute to the association between genetic variation in *Cacna1c* and psychiatric disorders.

## Supplementary Material

sbz127_suppl_Supplementary-Material
