# Supplementary material for: Cacna1c Hemizygosity Results in Aberrant Fear Conditioning to Neutral Stimuli
Source: Schizophr Bull. 2020 Jan 7;46(5):1231–8. doi: 10.1093/schbul/sbz127 (PMC7505182; doi:10.1093/schbul/sbz127)
Supplement: sbz127_suppl_Supplementary-Material [file sbz127_suppl_supplementary-material.doc]

**Supplementary Material**

**Supplemental Methods**

S1: Characterisation of *Cacna1c^+/-^* rats

*Cacna1c^+/-^* rats show approximately 50% *Cacna1c* mRNA reduction throughout the brain and a similar decrease in Ca_V_1.2 protein. Basic behaviour tasks revealed that there were no differences between *Cacna1c^+/-^*rats and wild-types in terms of locomotion or anxiety (Sykes et al, 2019). Genotypes were determined by PCR analysis (Fwd: 5’- GCTGCTGAGCCTTTTATTGG-3’; Rev: 5’-CCTCCTGGATAGCTGCTGAC-3’).

S2: Description of rat conditioning chambers used

Conditioning chambers were 32cm x 25.5cm x 27cm, with shock grid floors (19 stainless steel rods, 1 cm apart, 4.8mm diameter, Sandown Scientific, UK) above a stainless-steel pan. The chambers were enclosed within a second sound attenuating chamber with a ventilation fan providing background noise of 63db. Context A consisted of a non-illuminated box (with an infrared lamp to allow filming) with lavender scent (Aromatherapy Essential Oil, Boots). Context B contained fresh sawdust, star patterned walls and was lit by a house light (75W).

S3: Freezing following CS without associated footshock

There were no incidences of freezing behaviour from either wild-types or *Cacna1c^+/-^*rats when presented with the auditory cue in the absence of the associated US (p = 1) (Table S1)

Table S1: Freezing behaviour following CS presentation (Mean +/- SEM)

|  | Baseline (Freezing %) | Following CS presentation (Freezing %) |
| --- | --- | --- |
| Wild-types | 0 +/- 0 | 0 +/- 0 |
| *Cacna1c^+/-^* | 0 +/- 0 | 0 +/- 0 |
